# Dynamic forecasting and mechanisms of volatility synchronization in complex financial systems

**DOI:** 10.1371/journal.pone.0334853

**Published:** 2025-10-31

**Authors:** Jiang-Cheng Li, Jin Guo, Rui Ma, Guangyan Zhong

**Affiliations:** 1 School of Economics, Yunnan University of Finance and Economics, Kunming, People’s Republic of China; 2 School of Finance, Yunnan University of Finance and Economics, Kunming, People’s Republic of China; Roma Tre University: Universita degli Studi Roma Tre, ITALY

## Abstract

Synchronization, which has been a common natural phenomenon, occurs frequently in complex financial systems and is an important contagion mechanism for systemic financial risks and even financial crises. In view of this, we construct a coupled stochastic volatility model and its volatility synchronization analysis framework and combine machine learning methods and rolling cycle window to propose a prediction method for dynamic volatility synchronization. Taking the Shanghai Composite Index (SSEC) and Shenzhen Component Index (SZI) as binary synchronization examples, we analyze the dynamic forecasting performance of the proposed method in an in-sample and out-of-sample empirical comparison by combining multiple loss functions and Superior Predictive Ability (SPA) tests for high-frequency data. It is found that the in-sample estimates of our proposed model are highly consistent with the market behavior and that the model outperforms other models in predicting stock market volatility synchronization accuracy. In addition, by combining dynamic simulation with multivariate empirical mechanism analysis, our methodology not only explores synchronization dynamics but also identifies significant risk events, providing a comprehensive framework for understanding complex system behaviors.

## 1 Introduction

Synchronization is the collective behavior from two or more dynamically coupled units and is a natural phenomenon prevalent in natural systems [1]. The most successful attempt to study synchronization was made by the researcher Kuramoto in 1975, who proposed a model that describes the synchronization behavior of a large number of coupled oscillators and systematically established the foundational theory of synchronization [2], which laid the foundation for understanding the synchronization phenomenon in complex systems. Since then, with the development of complex network theory, researchers have begun to pay attention to the effect of network structure on synchronization behavior, forming a complex network synchronization theory [3]. In the early synchronization studies, scholars focused more on chaotic systems [4], complex networks [5], and so on. Nowadays, synchronization behavior has shown strong research value and application demand in various types of systems, attracting the attention of many scholars, including physics [6], biology [7], sociology [8,9], economics [10–13], engineering [14] and other fields. Similar to the synchronization effect in natural systems, various random variables in the financial market affect stock market volatility and may trigger synchronous behavior in stock market volatility [15]. Meanwhile, under economic globalization and financial integration, the transmission of local, regional, and international shocks is exacerbated. When a major event occurs, the financial market’s reaction mechanism can lead to rapid risk contagion among stock markets, i.e., a risk-synchronous behavior occurs [16,17]. Thus, the synchronized behavior of the financial system is worthy of in-depth analysis.

However, unlike the synchronous system described by the Kuramoto model, as a complex dynamic system, the financial system typically exhibits many complex dynamic behavioral characteristics [18]. Researchers have developed many models of stock price dynamics in this field in order to describe the dynamic behavior of stock market prices, such as the Geometric Brownian Motion Model [19], the GARCH Model [20], the Heston Model [21], etc. Among them, the Heston model, which consists of two coupled stochastic differential equations, well describes the statistical characteristics of stock prices. Various improved Heston models have also been widely used to simulate the dynamic process of stock prices and volatility in financial markets. For instance, the mean escape time in the improved Heston model based on the monostable potential function [22], the stabilizing effect of financial market volatility in the generalized Heston model [23], and the simulation of the dynamic behavior of the stock market during the COVID-19 period [24], etc. This also makes it difficult to analyze the synchronized behavior of the stock market using the Kuramoto method. Currently, for the analysis of stock market synchronization, scholars also often use stock price synchronization indicators based on decidable coefficients to portray the synchronization behavior in resonance effects. For example, Roll proposed the concept of stock price synchronization [25], Morck proposed a measure of stock return synchronization [10], and so on. The concept of volatility as a measure of risk in modern financial theory is widely accepted in finance [26]. Volatility has been extensively studied and applied [27], in which many stock market price dynamics models are constructed on the basis of volatility [19–21]. Moreover, with the gradual maturation of stock forecasting research, the majority of previous scholars’ literature focuses on the prediction studies of stock prices [28] and volatility [29,30]. However, while there are many factors currently inducing risky outbreaks of stock market volatility, the main manifestation of the risk is the synchronized behavior of amplified volatility. Therefore, this paper analyzes the price volatility synchronization between stock markets and then proposes a model of volatility synchronization, which is particularly important to extend the theory of synchronization, dynamics theory, and dynamic prediction of volatility synchronization in complex financial markets.

Consequently, based on the foundation of previous research and an in-depth discussion of stock market volatility synchronization, we plan to analyze the following issues: (i) How can the dynamic volatility synchronicity of the stock market be measured? (ii) What is the dynamic mechanism behind stock market volatility synchronization? (iii) How should one dynamically predict stock market volatility synchronicity? To address these questions, this paper attempts to make beneficial additions based on existing studies. Firstly, we introduce realized volatility as a measure of dynamic volatility synchronization in stock markets by integrating the concepts of stock price synchronization and stock return synchronization indexes proposed by previous scholars; secondly, we simulate the synchronization problem of volatility among different stock markets by using the coupled Heston model; and lastly, Our dynamic forecasting approach integrates machine learning techniques,showcasing the ability of the Heston model in achieving high consistency in predictions. This research distinguishes itself from previous studies in the following ways:

(i) A dynamic volatility synchronization method is proposed based on the concept of synchronization in econophysics, leveraging high-frequency data to enhance the efficiency of volatility estimation. Intraday fluctuations of high-frequency prices are less affected by measurement errors than low-frequency observations and that high-frequency data are more effective in estimating and predicting asset price volatility. Daily realized volatility (RV) is defined as the sum of intraday squared returns [31,32], which is considered a more efficient estimate of volatility than daily squared returns [33,34];

(ii) A coupled Heston model is proposed based on the Heston model, which reveals the dynamical evolution mechanism of stock market volatility synchronization. Volatility models of the stock market are one of the common methods used to describe the fluctuations in the stock market. The coupled Heston model we propose takes into account the fact that the correlated synchronized behavior between stock markets primarily originates from volatility, improving our understanding of the dynamic characteristics of complex financial systems;

(iii) Combining machine learning methods and adopting the rolling time window technique [35,36], we carry out dynamic forecasting of the coupled Heston model and obtain predicted volatility synchronization sequence data from the predicted volatility series data. At present, the literature studies of predecessors are mainly focused on price prediction models such as ARCH model, GARCH model, Stochastic Volatility model, and their derived models, but the Heston model exhibits the characteristic of high consistency between predicted data and actual data.

The rest of the paper is structured as follows. Sect 2 describes the coupled Heston model, volatility synchronization indicators, and the maximum likelihood method. Sect 3 focuses on out-of-sample forecasting and methods for evaluating forecasting effectiveness. Sect 4 presents a data description, results of volatility synchronization measurement, in-sample fitting results, and out-of-sample prediction results. Sect 5 is part of the analysis of the fluctuation synchronization dynamics mechanism. A brief discussion is given in Sect 6.

## 2 Methodology

### 2.1 Coupled Heston volatility model

The Heston model and its extended models are commonly used to describe the dynamic evolution characteristics of stock prices [21–24]. We extend the Heston model to describe the dynamic processes between *N* stock indices. The model is given by the following coupled stochastic differential equations:

$$\{dx_{i}(t)=-\frac{v_{i}(t)}{2}dt+\sqrt{v_{i}(t)}dZ_{i}\;dv_{i}(t)=\kappa_{i}(\theta_{i}-v_{i}(t))dt+\delta_{i}\sqrt{v_{i}(t)}dY_{i}\;\text{for }i=1,2,\ldots ,N$$ (1)

Where *x*_*i*_(*t*) and $v_{i}(t)$ are the logarithmic price and realized volatility of the *i* -th stock price index at time *t*, $\kappa_{i}$ is the mean reversion rate of the stock price index’s volatility $v_{i}(t)$, $\theta_{i}$ is the long-term mean of volatility $v_{i}(t)$, $\delta_{i}$ is the fluctuations of volatility, and *dt* is the time step. *dZ*_*i*_ and *dY*_*i*_ are correlated Wiener processes with the following statistical properties:

$$\{\left\langle dZ_{i}\right\rangle =\left\langle dY_{i}\right\rangle =0,\;\left\langle dZ_{i}(t)dY_{i}(t^{\prime })\right\rangle =\rho_{i}dt\delta (t-t^{\prime }),\;\left\langle dZ_{i}(t)dZ_{j}(t^{\prime })\right\rangle =0,\;\left\langle dY_{i}(t)dY_{j}(t^{\prime })\right\rangle =\rho_{i,j}dt\delta (t-t^{\prime })\;\text{for }i\neq j\;\text{for }i,j=1,2,\ldots ,N$$ (2)

here, $\rho_{i}$ quantifies the correlation between the *i*-th stock market’s self-volatility and prices, and $\rho_{i,j}$ considers the correlated synchronization behaviors between the *i*-th and *j*-th stock markets stemming mainly from volatility.

### 2.2 Volatility synchronization indicator

Unlike correlation analysis, which only reveals the linear relationship between two variables, synchronization accounts for nonlinear dynamic phenomena such as market resonance and herd effects. Notably, Roll utilized the goodness-of-fit measure *R*^2^ in the regression model to quantify the contribution of market factors to stock returns [25], and Morck transformed the goodness-of-fit *R*^2^ accordingly to calculate the synchronicity of stock prices [10]. We will continue to refer to Morck’s research methodology [10]. By introducing the realized volatility estimated based on 5-minute high-frequency data and regressing the volatilities of two stock market indices, we obtain the fitted coefficients *R*^2^, which are logarithmically deformed to derive the volatility synchronization indicator:

$$SYNCH_{i,j}=\ln\left(\frac{R_{i,j}^{2}}{1-R_{i,j}^{2}}\right)$$ (3)

$$R_{i,j}^{2}=(\rho_{v_{i},v_{j}}{)}^{2}$$ (4)

$v_{i}$ and $v_{j}$ (i≠j) represent the realized volatilities of the two stock market indices. The correlation coefficient *R*_*i*,*j*_ is a statistic that measures the strength of linear correlation between two markets. The value of $R_{i,j}^{2}$ ranges from 0 to 1, and the closer it is to 1, the stronger the linear correlation between the two variables. Also, the larger the value of *SYNCH*_*i*,*j*_, the stronger the volatility synchronization between the two stock markets. According to previous studies, the average test accuracies of the dynamic modeling methods using rolling time windows are all significantly higher than those of the static models [35,36].

### 2.3 Double coupled Heston volatility model and its dynamic synchronization

When considering only two markets or two stocks, based on the system of Eq (1), the double-coupled system can be transformed into the following coupled stochastic differential equations:

$$\{dx_{i}(t)=-\frac{v_{i}(t)}{2}dt+\sqrt{v_{i}(t)}dZ_{i},\;dv_{i}(t)=\kappa_{i}(\theta_{i}-v_{i}(t))dt+\delta_{i}\sqrt{v_{i}(t)}dY_{i}$$ (5)

Where *x*_*i*_(*t*) and $v_{i}(t)$ (i=1,2) are the logarithmic price and realized volatility of the *i*-th stock price index at time *t*, $\kappa_{i}$ is the mean reversion rate of the stock price index’s volatility $v_{i}(t)$, $\theta_{i}$ is the long-term mean of volatility $v_{i}(t)$, $\delta_{i}$ is the fluctuations of volatility, representing the magnitude of the rise and fall in volatility, and *dt* is the time step. *dZ*_*i*_ and *dY*_*i*_ are correlated Wiener processes with the following statistical properties:

$$\{\left\langle dZ_{i}\right\rangle =\left\langle dY_{i}\right\rangle =0,\;\left\langle dZ_{1}(t)dY_{1}(t^{\prime })\right\rangle =\rho_{1}dt\delta (t-t^{\prime }),\;\left\langle dZ_{2}(t)dY_{2}(t^{\prime })\right\rangle =\rho_{2}dt\delta (t-t^{\prime }),\;\left\langle dY_{1}(t)dY_{2}(t^{\prime })\right\rangle =\rho_{1,2}dt\delta (t-t^{\prime })$$ (6)

$\rho_{1}$ and $\rho_{2}$ quantify the correlation between the two stock markets’ self-volatility and prices, and $\rho_{1,2}$ considers the correlated synchronization behaviors between the two stock markets stemming mainly from volatility. These equations describe the coupling relationship between stock prices and volatility. Changes in volatility affect changes in asset prices, and vice versa.

By introducing the realized volatility estimated based on 5-minute high-frequency data and regressing the volatilities of two stock market indices, we obtain the fitted coefficients *R*^2^, which are logarithmically deformed to derive the volatility synchronization indicator:

$$\text{SYNCH}=\ln\left(\frac{R^{2}}{1-R^{2}}\right)$$ (7)

$$R^{2}=(\rho_{v_{1},v_{2}}{)}^{2}$$ (8)

$v_{1}$ and $v_{2}$ represent the realized volatilities of the two stock market indices. According to previous studies, the average test accuracies of the dynamic modeling methods using rolling time windows are all significantly higher than those of the static models [35,36]. Therefore, based on the above volatility synchronization indicator, we introduce the rolling time window technique to establish a dynamic volatility synchronization indicator:

$${\text{SYNCH}}_{t}=\ln\left(\frac{R_{t}^{2}}{1-R_{t}^{2}}\right)$$ (9)

*R*_*t*_ denotes the coefficient of determination (*R*^2^) computed from the regression of realized volatilities of stock indices within a rolling time window.

### 2.4 Parameter estimation method

There are many methods for estimating model parameters, such as the likelihood function [37–39], Bayesian estimation [40,41], and so on. The maximum likelihood function estimation method is one of the traditional and commonly used methods [37–39]. In this paper, in order to discuss the problem of parameter estimation for the model, we first based on a sample set:

$$\{x_{i}=\left\{x_{i,1},x_{i,2},\ldots ,x_{i,t},\ldots ,x_{i,N}\right\}\;v_{i}=\left\{v_{i,1},v_{i,2},\ldots ,v_{i,t},\ldots ,v_{i,N}\right\}$$ (10)

To discretize the stochastic model, the Eq (5) is differenced as follows, The parameters involved in the formula have been defined and specifically introduced in Sect 2.3 above.

$$\{x_{1,t+1}=x_{1,t}-\frac{v_{1,t}}{2}\Delta t+\sqrt{v_{1,t}}\sqrt{\Delta t}Z_{1}\;v_{1,t+1}=v_{1,t}+\kappa_{1}\left(\theta_{1}-v_{1,t}\right)\Delta t+\delta_{1}\sqrt{v_{1,t}}\sqrt{\Delta t}Y_{1}\;x_{2,t+1}=x_{2,t}-\frac{v_{2,t}}{2}\Delta t+\sqrt{v_{2,t}}\sqrt{\Delta t}Z_{2}\;v_{2,t+1}=v_{2,t}+\kappa_{2}\left(\theta_{2}-v_{2,t}\right)\Delta t+\delta_{2}\sqrt{v_{2,t}}\sqrt{\Delta t}Y_{2}$$ (11)

Then the likelihood function can be expressed as:

$$L\left(\phi |X\right)=\prod_{t=1}^{N}f_{\mu ,\Sigma }(x_{t})=\prod_{t=1}^{N}\frac{1}{(2\pi {)}^{2}}\frac{1}{|\Sigma {|}^{\frac{1}{2}}}e^{-\frac{1}{2}{\left(x_{t}-\mu \right)}^{T}\Sigma^{-1}\left(x_{t}-\mu \right)}$$ (12)

The final set of unknown parameters can be denoted as $\phi =\left(\kappa_{1},\theta_{1},\delta_{1},\kappa_{2},\theta_{2},\delta_{2},\rho_{1},\rho_{2},\delta_{1,2}\right)$. The values of these parameters are obtained by maximizing the log-likelihood function under the conditions specified above. Where,


$$x_{t}=\left[x_{1,t+1},v_{1,t+1},x_{2,t+1},v_{2,t+1}\right]$$

$$\mu =[\begin{array}{c} x_{1,t}-\frac{v_{1,t}}{2}\Delta t\\ v_{1,t}+\kappa_{1}\left(\theta_{1}-v_{1,t}\right)\Delta t\\ x_{2,t}-\frac{v_{2,t}}{2}\Delta t\\ v_{2,t}+\kappa_{2}\left(\theta_{2}-v_{2,t}\right)\Delta t \end{array}]$$

$$\Sigma_{t}=\Sigma \Delta t=[\begin{array}{cccc} v_{1,t} & \rho_{1}\delta_{1}v_{1,t} & 0 & 0\\ \rho_{1}\delta_{1}v_{1,t} & \delta_{1}^{2}v_{1,t} & 0 & \rho_{1,2}\delta_{1}\delta_{2}\sqrt{v_{1,t}v_{2,t}}\\ 0 & 0 & v_{2,t} & \rho_{2}\delta_{2}v_{2,t}\\ 0 & \rho_{1,2}\delta_{1}\delta_{2}\sqrt{v_{1,t}v_{2,t}} & \rho_{2}\delta_{2}v_{2,t} & \delta_{2}^{2}v_{2,t} \end{array}]\Delta t.$$

The expression


$$\max_{\phi }\log L\left(\phi |X\right)$$

signifies that our objective is to maximize the log-likelihood function in order to obtain the optimal estimates for the parameter *ϕ*. Meanwhile $f_{\mu ,\Sigma }(x_{t})$ denotes the probability density function of a multivariate Gaussian distribution, Next, we can utilize this distribution for parameter estimation.

$$logL\left(\phi |X\right)\propto -\frac{1}{2}\sum_{t=1}^{N}|\Sigma_{t}|\Delta t\;-\frac{1}{2}\sum_{t=1}^{N}{\left(x_{t}-\mu \right)}^{T}\Sigma_{t}^{-1}\left(x_{t}-\mu \right)\;\propto -\frac{1}{\Delta t}\sum_{t=1}^{N}\left[|\Sigma |\Delta t^{2}+{\left(x_{t}-\mu \right)}^{T}\Sigma^{-1}\left(x_{t}-\mu \right)\right]$$ (13)

$$logL\left(\phi |X\right)\propto \delta_{1}^{2}\delta_{2}^{2}\left(1-\rho_{1}^{2}-\rho_{1,2}^{2}-\rho_{2}^{2}+\rho_{1}^{2}\rho_{2}^{2}\right)\Delta t^{2}\sum_{t=1}^{N}[v_{1}^{2}v_{2}^{2}]\;+\frac{1}{1-\rho_{1}^{2}-\rho_{1,2}^{2}-\rho_{2}^{2}+\rho_{1}^{2}\rho_{2}^{2}}\;\times \sum_{t=1}^{N}[\frac{1-\rho_{1,2}^{2}-\rho_{2}^{2}}{v_{1}}\Delta x_{1v,t+1}^{2}\;-\frac{2\rho_{1}(1-\rho_{2}^{2})}{\delta_{1}v_{1}}\Delta x_{1v,t+1}\left(\Delta v_{1,t+1}-\kappa_{1}(\theta_{1}-v_{1,t})\Delta t\right)\;-\frac{2\rho_{1}\rho_{1,2}\rho_{2}}{\sqrt{v_{1}v_{2}}}\Delta x_{1v,t+1}\Delta x_{2v,t+1}\;+\frac{2\rho_{1}\rho_{1,2}}{\delta_{2}\sqrt{v_{1}v_{2}}}\Delta x_{1v,t+1}\left(\Delta v_{2,t+1}-\kappa_{2}(\theta_{2}-v_{2,t})\Delta t\right)\;+\frac{1-\rho_{1}^{2}}{\delta_{1}^{2}v_{1}}{\left(\Delta v_{1,t+1}-\kappa_{1}(\theta_{1}-v_{1,t})\Delta t\right)}^{2}\;-\frac{2\rho_{1,2}}{\delta_{1}\sqrt{v_{1}v_{2}}}\left(\Delta v_{1,t+1}-\kappa_{1}(\theta_{1}-v_{1,t})\Delta t\right)\Delta x_{2v,t+1}\;+\frac{2}{\delta_{1}^{2}\sqrt{v_{1}v_{2}}}\left(\Delta v_{1,t+1}-\kappa_{1}(\theta_{1}-v_{1,t})\Delta t\right)\left(\Delta v_{2,t+1}-\kappa_{2}(\theta_{2}-v_{2,t})\Delta t\right)\;+\frac{1-\rho_{1}^{2}}{\delta_{1}\delta_{2}v_{1}^{2}}\Delta x_{2v,t+1}^{2}\;+\frac{2(1-\rho_{1}^{2})}{v_{2}}\Delta x_{2v,t+1}\left(\Delta v_{2,t+1}-\kappa_{2}(\theta_{2}-v_{2,t})\Delta t\right)\;+\frac{\left(1-\rho_{1}^{2}\right)}{\delta_{1}^{2}v_{1}^{2}v_{2}}{\left(\Delta v_{2,t+1}-\kappa_{2}(\theta_{2}-v_{2,t})\Delta t\right)}^{2}]$$ (14)

The above two formulas respectively represent the general form of the log-likelihood function under the multivariate Gaussian distribution and its specific expansion under this model. The first formula provides the general expression of the log-likelihood, while the second formula fully expands it based on the model parameters and variables, clarifying the contribution of each parameter to the likelihood function. By maximizing this log-likelihood function, the parameter *ϕ* can be effectively estimated. Estimated parameters (e.g., $\kappa_{1},\theta_{1},\delta_{1},\kappa_{2},\theta_{2},\delta_{2},\rho_{1},\rho_{2},\delta_{1,2}$) are inferred via maximum likelihood estimation unless otherwise stated; observable quantities (e.g., *x*_*i*_(*t*) and $v_{i}(t)$) are derived from market data. In addition, the parameters can also be obtained by calculating the minimum variance between the model and actual data through simplex computation.

## 3 Forecasting methods

### 3.1 Rolling time window forecasting method

This paper further analyzes the rationality and predictive ability of the proposed model. For comparison, we also discuss the ARCH model, the GARCH (1,1) model, and the SV model. Based on the machine learning methods, out-of-sample daily volatility forecasting using rolling time windows is conducted for the coupled Heston model proposed in this paper and the three comparative models mentioned above. Subsequently, Constructing a volatility synchronization index based on dual-market volatility prediction sequences. We apply a standard machine learning framework in which data are divided into training and prediction (or test) samples, and the model is trained to forecast volatility synchronization. Our data ranges from January 2, 2003, to December 29, 2023, with 5100 realized volatility series data.

By dividing the data samples (t = 1, 2,..., N = 5100) into two parts, the estimation sample and the prediction sample[36,42–44].The estimation sample contains data for H = 1000 trading days and the prediction sample includes data for M = 4100 trading days after the estimation sample (i.e., t = H + 1, H + 2, ..., H + M). The forecast horizon is one-day-ahead. The prediction method is constructed as follows:This study adopts the rolling window dynamic prediction method for volatility prediction, with its core logic being updating samples through “fixed window + periodic sliding” to generate predictions. Specifically, the method takes as input a time-series data matrix **X** (e.g., daily stock returns of enterprises), an initial window size H=1000, and total forecast days *M*, and outputs a predicted volatility sequence $S^{\prime }$ with length *M*; in operation, it first initializes an empty vector $S^{\prime }$ to store results, then conducts *M* rounds of cyclic prediction. This method features no data leakage (only using historical data before the forecast date), dynamic adaptability (sliding window incorporating the latest information), and stability with flexibility.

### 3.2 Forecast evaluation

With the predicted series of volatility synchronization values $S_{m}^{\prime }$ for the coupled Heston model proposed in this paper, along with the three comparative models described above, we can assess the deviation of these forecast values from the standard of real market volatility estimation, denoted as *S*_*m*_ (where *S*_*m*_ represents the RV data) [45,46]. Currently, academics are not clear on which loss function is the most reasonable standard for measuring predictive deviation [36,42–44]. Therefore, Hansen and Lunde suggest that as many different forms of loss functions as possible can be used as criteria for judging the accuracy of forecasting models [47]. Based on this, this paper adopts five widely used statistical loss functions to evaluate the prediction accuracy of various types of volatility models separately.

The five loss functions are labeled as *Loss*_*i*_ (i = 1, 2,..., 5), among which *Loss*_1_ and *Loss*_2_ are respectively called the Mean Squared Error (MSE) and the Mean Absolute Error (MAE), which are the two most commonly used forms of loss functions in such judgment. *Loss*_3_, *Loss*_4_, and *Loss*_5_ are specifically the Mean Absolute Percentage Error (MAPE), Mean Squared Percentage Error (MSPE), and Root Mean Squared Error (RMSE). The specific definitions of each type of loss function are as follows [36,48]:


$$Loss_{1}:MAE\;=\;M^{-1}\sum_{H+1}^{H+M}|S_{m}-S_{m}^{\prime }|,$$

$$Loss_{2}:MSE\;=\;M^{-1}\sum_{H+1}^{H+M}(S_{m}-S_{m}^{\prime }{)}^{2},$$

$$Loss_{3}:MAPE\;=\;M^{-1}\sum_{H+1}^{H+M}|\frac{S_{m}-S_{m}^{\prime }}{S_{m}}|,$$

$$Loss_{4}:MSPE\;=\;M^{-1}\sum_{H+1}^{H+M}[\frac{S_{m}-S_{m}^{\prime }}{S_{m}}{]}^{2},$$

$$Loss_{5}:RMSE\;=\;\sqrt{M^{-1}\sum_{H+1}^{H+M}(S_{m}-S_{m}^{\prime }{)}^{2}},$$

Where *M* is the length of the forecast set. However, relying simply on some loss functions as the evaluation criteria for comparing the prediction accuracy of the models, may lead to conclusions that are not robust. Therefore, it is necessary to further enhance the reliability of the results through some statistical tests. Hansen and Lunde proposed a so-called “Superior Predictive Ability (SPA) test" [47], which employs a bootstrap method for simulation [42,44]. The SPA test has superior model discrimination capability than the similarity Reality Check (RC) test proposed by White [49], and the conclusions drawn from the SPA test are more robust. In other words, compared with other tests based on a single sample, the test conclusions obtained from the SPA test are more reliable, and its findings can be generalized to other similar data samples [50,51].

## 4 Empirical comparison

### 4.1 Data

The overall volatility risk of a stock market is typically reflected by the volatility of that stock market index. In this paper, the daily realized volatility (RV) series data calculated from 5-minute high-frequency data of two stock market indices, the Shanghai Securities Composite Index (SSEC) and the Shenzhen Securities Component Index (SZI), are proposed to be selected as the research samples. Moreover, the daily closing price and daily realized volatility data samples of these two stock market indices are sourced from the CSMAR database, covering the period from January 2, 2003, to December 29, 2023, resulting in a total of 5,100 trading days of data. In this case, the calculation method for the daily Realized Volatility in the CSMAR database refers to the literature of Wei, Li, and Chen, which defines it as the sum of the squared logarithmic returns of every 5-minute trading data [45,46].

The descriptive statistics of the log prices, log returns, and realized volatilities of the SSEC and the SZI are given in Table 1. From Table 1, it can be observed that the realized volatility series of the market indices exhibit characteristics of sharp peaks and thick tails, which indicates that the volatility of both indices is quite drastic and far beyond the range assumed by the normal distribution (the Jarque-Bera statistic is significant in both cases). Meanwhile, the logarithmic price *x*_*i*_, logarithmic return *r*_*i*_, and realized volatility $v_{i}$ series data demonstrate very significant autocorrelation characteristics over a very long time horizon, suggesting persistent volatility with long-memory characteristics. Further, the ADF unit root test shows that the original hypothesis of the existence of unit root is significantly rejected for each series. Therefore, it can be considered that the individual series are smooth and thus can be further analyzed and modeled.

**Table 1:** Descriptive statistics of log prices ($x_{i}$), log returns ($r_{i}$), and realized volatility ($v_{i}$) of stock market indices.

	SSEC			SZI		
	$x_{1}$	$r_{1}$	$v_{1}$	$x_{2}$	$r_{2}$	$v_{2}$
Mean	3.4078	6.68e−05	0.0155	3.9351	0.0001	0.0205
Median	3.4587	0.0002	0.0074	4.0062	0.0002	0.0115
Max	3.7848	0.0392	0.4266	4.2907	0.0398	0.6045
Min	3.0050	−0.0402	1.16e−05	3.4186	−0.0423	3.40e−07
St.Dev.	0.1529	0.0065	0.0263	0.2145	0.0075	0.0316
Skewness	−0.6776	−0.5269	6.2552	−1.0834	−0.4610	6.3313
Kurtosis	−0.0446	5.0840	60.8180	0.0400	3.3775	65.416
J−B	390.43^***^	5715.14^***^	817670.0^***^	997.37^***^	2598.17^***^	941585.6^***^
*Q* ^(5)^	25329.74^***^	19.58^***^	8482.34^***^	25344.32^***^	21.21^***^	7186.45^***^
*Q* ^(10)^	50357.15^***^	39.88^***^	12686.98^***^	50410.23^***^	34.59^***^	10398.25^***^
*Q* ^(20)^	99488.01^***^	70.07^***^	20475.31^***^	99695.26^***^	50.58^***^	16651.95^***^
ADF	−2.0874	−16.826^***^	−6.7862^***^	−2.2923	−25.972^***^	–6.5636^***^

Note: The symbols *, **, and *** represent significant at the 10%, 5%, and 1% levels, respectively, and *Q*^(*n*)^ is the Ljung-Box Q statistic of lag order *n*.

### 4.2 Analysis of volatility synchronization

To illustrate the volatility synchronization model of the stock market, this paper calculates the volatility synchronization values between the SSEC and the SZI over the full sample period (Sect 4.1), using Eqs (7) and (8). The actual synchronization value is found to be *SYNCH* = 2.1223.

**Fig 1:**
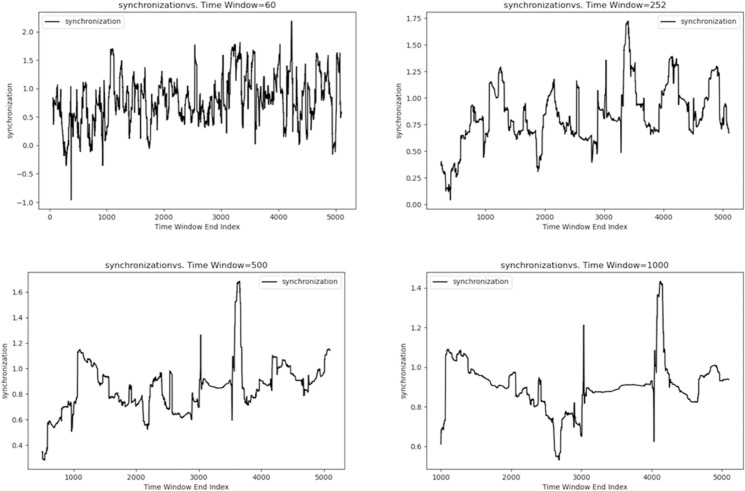
The dynamic volatility synchronization of the SSEC and the SZI at multiple scales (60, 252, 500, and 1000 days).

Additionally, to explore the volatility synchronization across stock markets at multiple scales, we conduct dynamic volatility synchronization simulations for the SSEC and the SZI at four scales (60, 252, 500, and 1,000 days) using the estimated parameters of the coupled Heston model. Fig 1 presents the results, showing significant synchronization between the two markets across all scales—with the highest synchronization observed at the 60-day (short-term) scale.

### 4.3 Comparison of in-sample fitting results

This paper selects the index price and volatility real data of the SSEC and the SZI to estimate the parameters of the model. We set the objective function to be the sum of the average mean squared errors between the coupled Heston model estimation sequence and the actual sequence. Then, we assign initial values to the parameters *ϕ* of the coupled Heston model. Subsequently, we iteratively use the Nelder-Mead method, a numerical optimization algorithm, to adjust the model parameters in order to minimize the value of the objective function. Finally, we identify the set of parameters *ϕ* that minimizes the objective function. Through the above steps, the estimated values of the parameters for the equation are obtained as follows: $\kappa_{1}\;=\;0.9908$, $\theta_{1}\;=\;0.0098$, $\sigma_{1}\;=\;0.0972$, $\rho_{1}\;=\;-0.1037$, $\kappa_{2}\;=\;1.0222$, $\theta_{2}\;=\;0.0101$, $\sigma_{2}\;=\;0.0994$, $\rho_{2}\;=\;-0.1037$, $\rho_{1,2}\;=\;-0.1037$.

**Fig 2:**
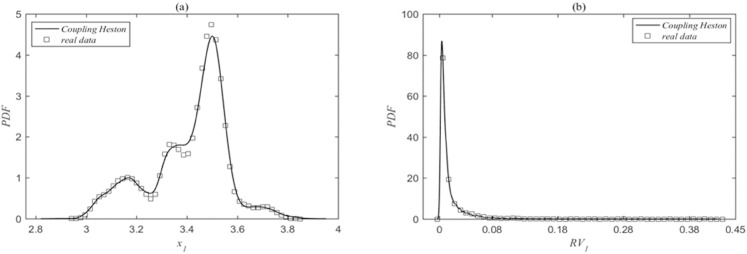
(a) and (b) are the probability density functions of the SSEC price and realized volatility. The squares denoting the real data and the solid lines indicating the model results. The parameter fitting results are: $\kappa_{1}\;=\;0.9908$, $\theta_{1}\;=\;0.0098$, $\sigma_{1}\;=\;0.0972$, $\rho_{1}\;=\;-0.1037$, $\kappa_{2}\;=\;1.0222$, $\theta_{2}\;=\;0.0101$, $\sigma_{2}\;=\;0.0994$, $\rho_{2}\;=\;-0.1037$, $\rho_{1,2}\;=\;-0.1037$.

**Fig 3:**
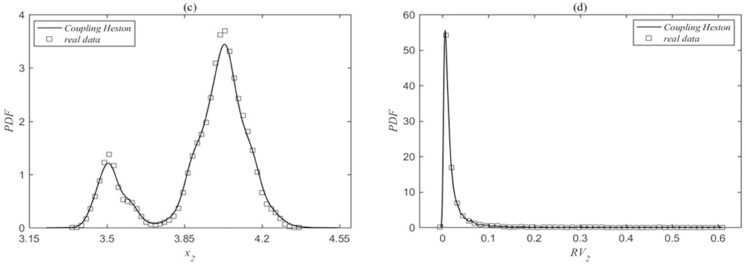
(c) and (d) are the probability density functions of the SZI price and realized volatility. The squares denoting the real data and the solid lines indicating the model results. The parameter values are consistent with those in Fig 2.

Then, we combined the serial data of parameter value estimation and the real data to calculate the probability density functions of prices and realized volatilities, as shown in Figs 2 and 3. The comparison between the real data (squares) and the model results (solid lines) for the SSEC and the SZI is presented in the figures. Fig 2a and 2b show the probability density functions of the index price and realized volatility for the SSEC, while Fig 3c and 3d represent those for the SZI, respectively. It is evident that the probability density functions of index prices and realized volatilities simulated by the coupled Heston model are in good agreement with the actual data results, indicating a good fit for the simulation outcomes. Meanwhile, based on the realized volatility series data obtained from the simulation, the simulated result volatility synchronization value is obtained as *SYNCH^model^* = 2.1063, and the real synchronization value is *SYNCH^data^* = 2.1223, which is a result that further demonstrates the reliability of the model.

### 4.4 Comparison of out-sample dynamic predictions

**Fig 4:**
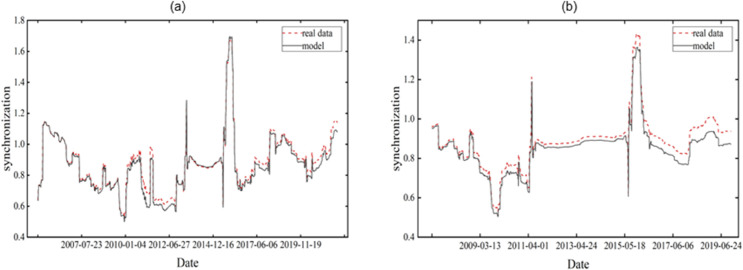
The out-of-sample rolling forecasting results of the coupled Heston model with different estimation sample intervals (H = 500 and 1000).

Fig 4a shows the dynamic volatility synchronization prediction results of the coupled Heston stock price dynamics model mentioned in the paper for the prediction sample interval t = 501, 502,..., 5100 (represented by the solid line), while the dynamic volatility synchronization results computed based on the real data are represented by the red dashed line. Similarly, Fig 4b shows the dynamic volatility synchronization prediction results of the stock market for the predicted sample interval t = 1001, 1002,..., 5100. As can be seen from the comparison of the figures, the prediction results of the proposed model in this paper align well with the actual results under different prediction sample intervals.

To explore the out-of-sample volatility synchronization predictive performance of the models proposed in this paper, we conducted a comparison of their out-of-sample prediction capabilities. Table 2 gives the results of the volatility synchronization value prediction test of various volatility models under five loss functions, where the definitions of the five loss functions *Loss*_*i*_ are shown in Sect 3.2. The results from the table show that:

**Table 2:** The loss function values for the synchronization prediction of various models.

	Coupled Heston model	ARCH model	GARCH model	SV model
MAE	0.03483	0.37348	0.39226	1.57248
MSE	0.00174	0.24628	0.19209	3.64023
MAPE	0.03923	0.44766	0.44485	1.76765
MSPE	0.00212	0.38916	0.24352	4.66848
RMSE	0.04170	0.49627	0.43828	1.90794

(i) The coupled Heston model attains the lowest loss across the evaluated loss functions and ranks first. This indicates that the model better predicts the dynamic volatility synchronization of the stock market, demonstrating superior synchronization value forecasting performance than other models.

(ii) As far as the comparison with other ARCH, GARCH, and SV models is concerned, the GARCH model is ranked second in terms of the loss functions MSE, MAPE, MSPE, and RMSE and has relatively good predictive performance, whereas the SV model achieves the lowest prediction accuracies across all loss functions.

However, further SPA testing of this prediction is necessary to obtain more robust and broadly applicable conclusions. The SPA test is a statistical method for comparing the predictive performance of multiple models, which evaluates whether a base model significantly outperforms a set of alternative models under a specified loss function.

Table 3 shows the SPA test results obtained after 10,000 bootstrap simulation processes. Column 1 of Table 3 represents the five loss functions *Loss*_*i*_, while column 2 lists the name of the model selected as the base model ($M_{i}(i\;=\;0,...,3)$ respectively denote the coupled Heston model, ARCH model, GARCH model, and SV model).The numbers in the table represent the p-values of the SPA test. Specifically, under a certain loss function *Loss*_*i*_ judgment criterion, if the SPA test p-value of the base model is larger (closer to 1) relative to the other models, it indicates that the base model is the best-performing forecast model. Conversely, if the p-value is smaller, it suggests that the base model exhibits inferior predictive performance relative to the comparison models. The results from the table show that:

**Table 3:** The SPA test values for predicting volatility synchronization for various models.

Loss	Basic model	Comparative model			
		$M_{0}$	$M_{1}$	$M_{2}$	$M_{3}$
MAE	*M*_0_	0	1.0000	1.0000	1.0000
	*M*_1_	0.5038	0	0.4989	1.0000
	*M*_2_	0.5030	1.0000	0	1.0000
	*M*_3_	0.5036	0.4990	0.5025	0
MSE	*M*_0_	0	1.0000	1.0000	1.0000
	*M*_1_	0.5041	0	0.4914	1.0000
	*M*_2_	0.4985	1.0000	0	1.0000
	*M*_3_	0.5028	0.4990	0.5033	0
MAPE	*M*_0_	0	1.0000	1.0000	1.0000
	*M*_1_	0.5018	0	0.5006	1.0000
	*M*_2_	0.5050	1.0000	0	1.0000
	*M*_3_	0.5035	0.5048	0.5036	0
MSPE	*M*_0_	0	1.0000	1.0000	1.0000
	*M*_1_	0.5004	0	0.5021	1.0000
	*M*_2_	0.5005	1.0000	0	1.0000
	*M*_3_	0.5087	0.4977	0.5093	0
RMSE	*M*_0_	0	1.0000	1.0000	1.0000
	*M*_1_	0.5068	0	0.4952	1.0000
	*M*_2_	0.5031	1.0000	0	1.0000
	*M*_3_	0.5042	0.5067	0.5045	0

(i) Using the SSEC and the SZI (representing two major stock markets in China) as examples, the SPA test results for the five loss functions all indicate that when the coupled Heston model proposed in this paper is used as the base model, the SPA test p-value is approximately equal to 1 relative to the other models. This suggests that the coupled Heston model, which considers that the correlated synchronization behavior between stock markets primarily originates from volatility, can better predict the dynamic volatility synchronization among the stock markets.

(ii) The SV model is the worst-performing forecasting model under almost all loss function criteria. Overall, the four models’ ability to predict dynamic volatility synchronization across stock markets ranks in the following order (from best to worst): the coupled Heston model outperforms the GARCH model, the GARCH model outperforms the ARCH model, and the ARCH model outperforms the SV model.

(iii) The conclusions drawn from the SPA test results in Table 3 are generally consistent with those obtained in Table 2, and thus these conclusions have considerable universality for the prediction of volatility synchronization among stock markets.

## 5 The dynamical mechanisms of synchronization

### 5.1 Synchronous correlation mechanism

In the previous sections, we conducted in-sample and out-of-sample empirical comparative analyses of the proposed method’s dynamic forecasting performance, using the Shanghai Composite Index and Shenzhen Component Index as examples, based on the coupled Heston model and its dynamic synchronization framework. The results demonstrate that the in-sample estimation of the proposed model aligns closely with market behavior and exhibits superior predictive performance, thereby addressing the research question of how to dynamically measure and forecast cross-market volatility synchronization. To explore the dynamical mechanisms underlying volatility synchronization, this paper will further investigate the dynamical mechanisms of volatility synchronization across markets by combining dynamic simulations and multivariate empirical mechanism analysis.

Upon scrutiny of the current model, it becomes evident that each synchronization metric derived from real data is intricately linked to a distinct set of estimated parameters within the framework of our proposed coupled model. The determination of each synchronization metric is contingent upon a spectrum of estimated parameters, including but not limited to $\rho_{1}$, $\rho_{2}$, and $\rho_{1,2}$. This realization paves the way for a detailed comparative analysis between the synchronization metrics and the respective parameters of the coupled model.

Our investigation will focus on identifying any discernible patterns that may exist between the oscillations in synchronization metrics and the alterations in these parameters. The ultimate goal is to unravel the dynamical mechanisms at play, providing a deeper understanding of the system’s behavior. Of particular interest is the pronounced connection between the synchronization metrics and the parameter $\rho_{1,2}$, as demonstrated by the following Eq (15). This relationship will be the focus of our examination, as we seek to elucidate the patterns of their fluctuations and impact on the dynamic mechanisms, thereby guiding our understanding of the synchronization process.

$$SYNCH_{t}^{model}=\ln\left(\frac{R_{t}^{2}}{1-R_{t}^{2}}\right)$$ (15)

In this study, our analysis is based on comprehensive datasets comprising 4,101 synchronization metrics, as previously obtained, along with their corresponding optimal parameter set: $\kappa_{1}$, *θ*_1_, $\sigma_{1}$, $\rho_{1}$, $\kappa_{2}$, *θ*_2_, $\sigma_{2}$, $\rho_{2}$, and $\rho_{1,2}$. A rolling window with an average span of 1,000 data points has been employed to smooth out short-term fluctuations and highlight longer-term trends. To ensure the date’s authenticity and reliability for our analysis, stringent filtering and normalization processes have been applied.

**Fig 5:**
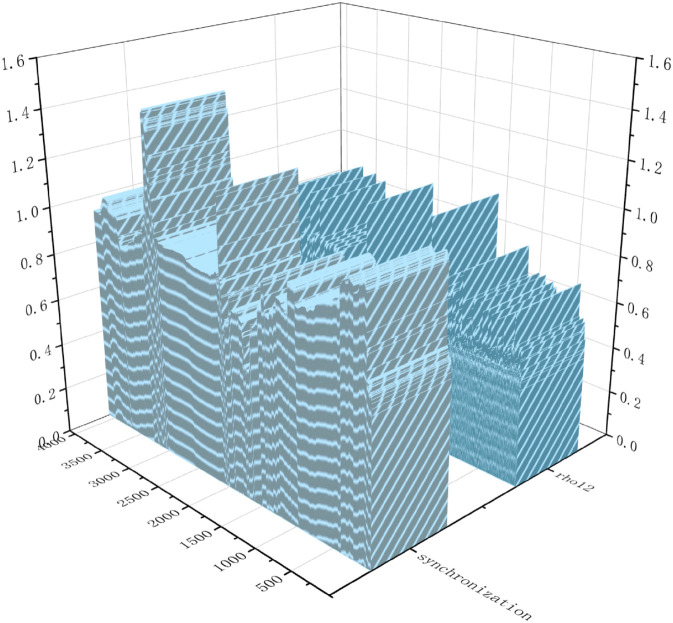
Comparison of synchronization metrics with $\rho_{1,2}$ frequencies.

Through comparative analysis, we have identified a notable correlation between the synchronization metrics and the parameter $\rho_{1,2}$. As illustrated in Fig 5, there is a striking similarity in the frequency characteristics between the synchronization metrics and the $\rho_{1,2}$ as proposed by our model. This is particularly evident in the synchronicity of peak occurrences, suggesting a dynamic interplay that warrants further investigation. Our findings underscore the significance of $\rho_{1,2}$ in understanding the underlying mechanisms that drive synchronization within the system.

The synchronicity in the occurrence of peaks within two sets of data may indicate an underlying shared dynamical mechanism or interplay between them. Such synchronization holds substantial significance within dynamical systems, as it may unveil the intrinsic coupling behaviors within the system. This suggests an interdependent, mutually influential, and regulatory relationship between the synchronization metrics and the parameter $\rho_{1,2}$. By delving into these studies, we can achieve a more profound comprehension of the internal generative mechanisms of synchronization metrics and how they respond to changes in external conditions. This understanding allows us to anticipate how synchronization metrics will evolve over time and adapt to various stimuli, providing a foundation for more effective management and decision-making in complex dynamic environments.

### 5.2 Multivariate analysis of synchronous dynamics mechanism

Table 4 provides descriptive statistical data for ten variables, including the mean, standard deviation (SD), first quartile (p25), median (p50, which is also the second quartile), and third quartile (p75). Based on this table, the following conclusions can be drawn: The data in the second row, namely the volatility synchronization metric, has a median (p50) of 0.906, which is close to the mean of 0.903, indicating that the volatility synchronization metric is generally high and the distribution is relatively symmetrical. Additionally, the first quartile (p25) and the third quartile (p75) are 0.860 and 0.957, respectively, showing the range of the middle of the synchronization metric data. The synchronization metric exhibits a certain standard deviation, suggesting that synchronization varies under different parameter conditions.

**Table 4:** Descriptive statistics of the main variables.

Variable	Mean	SD	p25	p50	p75
SYNCH	0.903	0.125	0.860	0.906	0.957
$\kappa_{1}$	1.005	0.014	1.000	1.003	1.011
*θ*_1_	0.010	0.000	0.010	0.010	0.010
$\sigma_{1}$	0.100	0.001	0.100	0.100	0.101
$\rho_{1}$	−0.100	0.001	−0.101	−0.100	−0.100
$\kappa_{2}$	1.005	0.014	1.000	1.002	1.011
*θ*_2_	0.010	0.000	0.010	0.010	0.010
$\sigma_{2}$	0.100	0.001	0.100	0.100	0.101
$\rho_{2}$	−0.100	0.001	−0.101	−0.100	−0.100
$\rho_{1,2}$	−0.101	0.001	−0.101	−0.100	−0.100

The data from rows 3 to 11 represent the main parameters of the coupling model, and the majority of them are very stable with minimal standard deviation, implying that these parameters exhibit consistent behavior within the studied system. The distribution of all data is close to a normal distribution, as the median is near the mean, and the interquartile range is relatively narrow. These parameters are used to describe the dynamical model, where the κ and *σ* parameters may be related to the system’s response characteristics. The stability of the *θ* parameters may indicate that their role in the model is fixed or unaffected by external conditions, while the *ρ* parameters may be associated with the strength of synchronization.

Based on the information mentioned above, we conducted a baseline regression analysis between the parameters in the coupling model and the synchronization metrics to further understand the impact of these model parameters on synchronization. The results are shown in Table 5, from which we can observe the following: $\kappa_{1}$ and $\kappa_{2}$ represent the mean reversion speeds of the volatility of stock price indices for two different stock markets. Their coefficients are −0.413 and −0.438, respectively, and are significant at the 0.05 significance level. This indicates that when the mean reversion speed of the volatility of the stock price indices in the stock markets increases, the synchronization between the two markets decreases. *θ*_1_ and *θ*_2_ indicate the long-term mean volatility of stock price indices for the two stock markets. The coefficient for *θ*_1_ is −36.106, significant at the 0.1 significance level, while the coefficient for *θ*_2_ is −11.265, which is not significant. This may imply that *θ*_1_ has a negative impact on synchronization, but the effect of *θ*_2_ is not significant.

**Table 5:** Baseline regression results of the synchronization metric with the model parameters.

$\kappa_{1}$	−0.413** (−2.22)
*θ*_1_	−36.106* (−1.95)
$\sigma_{1}$	−3.327* (−1.78)
$\rho_{1}$	3.071* (1.66)
$\kappa_{2}$	−0.438** (−2.34)
*θ*_2_	−11.265 (−0.61)
$\sigma_{2}$	−3.096 (−1.63)
$\rho_{2}$	3.171* (1.72)
$\rho_{1,2}$	3.167* (1.74)
Constant	3.825*** (3.01)

Note: The symbols *, **, and *** represent significant at the 10%, 5%, and 1% levels, and the data in the parentheses are t-value.

$\sigma_{1}$ and $\sigma_{2}$ denote the fluctuations in volatility of two stock markets. The coefficient for $\sigma_{1}$ is -3.327, significant at the 0.1 significance level, while the coefficient for $\sigma_{2}$ is −3.096, which is not significant. This suggests that $\sigma_{1}$ has a negative effect on synchronization, but the impact of $\sigma_{2}$ is not significant. $\rho_{1}$ and $\rho_{2}$ reflect the correlation between the volatility and prices within each of the two stock markets. Their coefficients are 3.071 and 3.171, respectively, and are also significant at the 0.1 significance level. This indicates that when the correlation between volatility and prices within the stock markets is stronger, synchronization also increases. $\rho_{1,2}$ manifests the primary source of the correlated synchronous behavior between the two stock markets. Its coefficient is 3.167, significant at the 0.1 significance level. This suggests that the correlated synchronous behavior between the two stock markets has a positive effect on synchronization.

### 5.3 Identification of significant extreme events based on volatility synchronization

**Fig 6:**
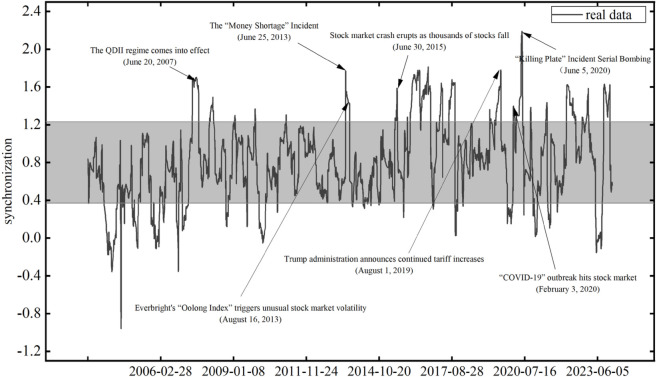
Volatility synchronization significant risk event identification chart. The reference lines for the gray area are the mean ± 1 standard deviation.

To further identify and detect significant risk events through extreme volatility synchronization, Fig 6 presents the identification chart of significant risk events in volatility synchronization on a 60-day scale. We can observe that after major risk events such as the “money crunch” in 2013, the stock market crash in 2015, the Trump administration’s announcement of continued tariff hikes on China in 2019, and the “COVID-19” pandemic in 2020, the peaks of dynamic volatility synchronization between the two stock markets, namely the Shanghai Composite Index and the Shenzhen Component Index, have identified these significant risk events.

Meanwhile, the measured volatility synchronization values when these identified significant risk events occurred are all above the sum of the mean and the standard deviation. This indicates that the two markets experienced high-intensity volatility synchronization simultaneously, revealing the linkage effect of volatility synchronization triggered by risk synchronization, where the risk occurring in one market spreads to the other market.

In addition, after the implementation of the QDII (Qualified Domestic Institutional Investor) system in 2007, it can be found that the volatility synchronization between the two markets has significantly increased. This conclusion is consistent with the statement that measures such as the liberalization of QFII (Qualified Foreign Institutional Investors) and QDII quotas have increased the connectivity between China and international capital markets and enhanced the linkage of fluctuations in China’s stock market.

Therefore, the dynamic volatility synchronization index constructed based on the realized volatility in this paper can effectively measure the volatility synchronization between stock markets, and significant risk events can be further identified through the peaks of volatility synchronization.

## 6 Conclusions

Synchronization phenomena, prevalent in natural systems, also play a crucial role in complex financial systems, particularly as a contagion mechanism for systemic financial risks and crises. This study aimed to address the dynamic synchronization of stock market volatility by proposing a coupled stochastic volatility model and a volatility synchronization analysis framework. We integrated machine learning concepts and rolling cycle windows to predict dynamic synchronization, focusing on the Shanghai Composite Index (SSEC) and Shenzhen Component Index (SZI) as case studies.

Therefore, this paper constructs a volatility synchronization index for portraying the risk synchronization between markets to portray the risk synchronization between markets by introducing realized volatility and proposes a coupled Heston model that considers the primary sources of volatility between stock markets based on previous studies. In addition, the integration of machine learning techniques with the proposed model set employs evolutionary mechanisms to dynamically predict stock market volatility synchronization using a rolling time window approach, and the dynamic predictive performance of the model proposed in this paper is discussed. Finally, the dynamics of volatility synchronization between markets are explored through a combination of dynamic simulation and multivariate empirical mechanism analysis. The results show that:

(i) The obvious high volatility synchronization between the SSEC Index and the SZI Index indicates that the two stock markets exhibit strong interconnectedness in their fluctuations, with a higher likelihood of risk synchronization occurring during significant unexpected events. (ii) The in-sample estimation results show that the estimated data are in good agreement with the real data, and the simulation results fit well, suggesting that the coupled Heston model proposed in this paper can effectively capture the dynamic mechanism of volatility synchronization between stock markets. (iii) The out-of-sample dynamic forecasting results highlight the superior forecasting ability of the coupled Heston model based on realized volatility over the ARCH, GARCH, and SV models based on daily return data under the multiple loss function criterion examined. (iv) Using the SPA test method proposed by Hansen and Lunde for determining the strengths and weaknesses of models, it is further confirmed that the coupled Heston model remains the most accurate model for forecasting volatility synchronization, followed by the GARCH model. (v) The proposed method can effectively identify significant financial market risk events through synchronization detection. This paper introduces the concept of synchronization in econophysics and provides a new research perspective for exploring the interconnections and risk contagion between stock markets from the viewpoint of stock market volatility synchronization. Simultaneously, the dynamic volatility synchronization measurement and forecasting analysis methods we have constructed help investors formulate and effectively adjust cross-market asset portfolio strategies, as well as provide more diverse research perspectives for financial regulators to prevent, manage, and regulate stock market volatility risks.

## References

[1] Wu X, Wu X, Wang C-Y, Mao B, Lu J, Lü J, et al. Synchronization in multiplex networks. Physics Reports. 2024;1060:1–54. doi: 10.1016/j.physrep.2024.01.005

[2] Acebrón JA, Bonilla LL, Pérez Vicente CJ, Ritort F, Spigler R. The Kuramoto model: a simple paradigm for synchronization phenomena. Rev Mod Phys. 2005;77(1):137–85. doi: 10.1103/revmodphys.77.137

[3] Rodrigues FA, Peron TKDM, Ji P, Kurths J. The Kuramoto model in complex networks. Physics Reports. 2016;610:1–98. doi: 10.1016/j.physrep.2015.10.008

[4] Voss H. Anticipating chaotic synchronization. Phys Rev E Stat Phys Plasmas Fluids Relat Interdiscip Topics. 2000;61(5A):5115–9. doi: 10.1103/physreve.61.5115 11031554

[5] Jalili M, Rad AA, Hasler M. Enhancing synchronizability of weighted dynamical networks using betweenness centrality. Phys Rev E Stat Nonlin Soft Matter Phys. 2008;78(1 Pt 2):016105. doi: 10.1103/PhysRevE.78.016105 18764018

[6] Huang Y, Huang H, Huang Y, Wang Y, Yu F, Yu B. Drive–response asymptotic shape synchronization for a class of two-dimensional chaotic systems and its application in image encryption. Physica D: Nonlinear Phenomena. 2024;463:134162. doi: 10.1016/j.physd.2024.134162

[7] Ott E, Antonsen TM Jr. Frequency and phase synchronization in large groups: low dimensional description of synchronized clapping, firefly flashing, and cricket chirping. Chaos. 2017;27(5):051101. doi: 10.1063/1.4983470 28576094

[8] Shahal S, Wurzberg A, Sibony I, Duadi H, Shniderman E, Weymouth D, et al. Synchronization of complex human networks. Nat Commun. 2020;11(1):3854. doi: 10.1038/s41467-020-17540-7 32782263 PMC7419301

[9] Lamontagne A, Legou T, Bedossa T, Gaunet F. Walk with me? Part 1: Dogs synchronize with an unfamiliar person who first synchronized with them. Applied Animal Behaviour Science. 2024;272:106204. doi: 10.1016/j.applanim.2024.106204

[10] Morck R, Yeung B, Yu W. The information content of stock markets: why do emerging markets have synchronous stock price movements?. Journal of Financial Economics. 2000;58(1–2):215–60. doi: 10.1016/s0304-405x(00)00071-4

[11] Saavedra S, Hagerty K, Uzzi B. Synchronicity, instant messaging, and performance among financial traders. Proc Natl Acad Sci U S A. 2011;108(13):5296–301. doi: 10.1073/pnas.1018462108 21402941 PMC3069203

[12] Gao H-L, Li J-C, Guo W, Mei D-C. The synchronicity between the stock and the stock index via information in market. Physica A: Statistical Mechanics and its Applications. 2018;492:1382–8. doi: 10.1016/j.physa.2017.11.065

[13] Crespo Cuaresma J, Fernández O. Explaining long-term bond yields synchronization dynamics in Europe. Economic Modelling. 2024;133:106684. doi: 10.1016/j.econmod.2024.106684

[14] Liang X, Zhang Y, Li D, Ge SS, How BVE, Lee TH. Synchronized tracking control for dynamic positioning vessel. Intl J Robust & Nonlinear. 2023;34(1):270–95. doi: 10.1002/rnc.6970

[15] Tao C, Zhong G-Y, Li J-C. Dynamic correlation and risk resonance among industries of Chinese stock market: New evidence from time–frequency domain and complex network perspectives. Physica A: Statistical Mechanics and its Applications. 2023;614:128558. doi: 10.1016/j.physa.2023.128558

[16] Cifuentes R, Ferrucci G, Shin HS. Liquidity risk and contagion. Journal of the European Economic Association. 2005;3(2–3):556–66. doi: 10.1162/jeea.2005.3.2-3.556

[17] Tiwari AK, Aikins Abakah EJ, Gabauer D, Dwumfour RA. Dynamic spillover effects among green bond, renewable energy stocks and carbon markets during COVID-19 pandemic: implications for hedging and investments strategies. Glob Financ J. 2022;51:100692. doi: 10.1016/j.gfj.2021.100692 38013879 PMC9761843

[18] Zhou W, Zhong G-Y, Leng N, Li J-C, Xiong D-P. Dynamic behaviors and measurements of financial market crash rate. Physica A: Statistical Mechanics and its Applications. 2019;527:121427. doi: 10.1016/j.physa.2019.121427

[19] Mantegna RN, Stanley HE. Introduction to econophysics: correlations and complexity in finance. Cambridge University Press; 1999.

[20] Bollerslev T. Generalized autoregressive conditional heteroskedasticity. Journal of Econometrics. 1986;31(3):307–27. doi: 10.1016/0304-4076(86)90063-1

[21] Heston SL. A closed-form solution for options with stochastic volatility with applications to bond and currency options. Rev Financ Stud. 1993;6(2):327–43. doi: 10.1093/rfs/6.2.327

[22] Bonanno G, Valenti D, Spagnolo B. Mean escape time in a system with stochastic volatility. Phys Rev E Stat Nonlin Soft Matter Phys. 2007;75(1 Pt 2):016106. doi: 10.1103/PhysRevE.75.016106 17358223

[23] Valenti D, Fazio G, Spagnolo B. Stabilizing effect of volatility in financial markets. Phys Rev E. 2018;97(6–1):062307. doi: 10.1103/PhysRevE.97.062307 30011541

[24] Jin L, Zheng B, Ma J, Zhang J, Xiong L, Jiang X, et al. Empirical study and model simulation of global stock market dynamics during COVID-19. Chaos Solitons Fractals. 2022;159:112138. doi: 10.1016/j.chaos.2022.112138 35493400 PMC9040430

[25] Roll R. R2. The Journal of Finance. 1988;43(3):541–66.

[26] Markowits HM. Portfolio selection. Journal of Finance. 1952;7(1):71–91.

[27] Dwyer GP, Hafer RW. The stock market: bubbles, volatility, and chaos. Springer; 2013.

[28] Henriques I, Sadorsky P. Forecasting rare earth stock prices with machine learning. Resources Policy. 2023;86:104248. doi: 10.1016/j.resourpol.2023.104248

[29] Liu J, Ma F, Zhang Y. Forecasting the Chinese stock volatility across global stock markets. Physica A: Statistical Mechanics and its Applications. 2019;525:466–77. doi: 10.1016/j.physa.2019.03.097

[30] Chen Y, Qiao G, Zhang F. Oil price volatility forecasting: threshold effect from stock market volatility. Technological Forecasting and Social Change. 2022;180:121704. doi: 10.1016/j.techfore.2022.121704

[31] Andersen TG, Bollerslev T, Diebold FX, Labys P. The distribution of realized exchange rate volatility. Journal of the American Statistical Association. 2001;96(453):42–55. doi: 10.1198/016214501750332965

[32] Andersen TG, Bollerslev T, Diebold FX, Labys P. Modeling and forecasting realized volatility. Econometrica. 2003;71(2):579–625. doi: 10.1111/1468-0262.00418

[33] McAleer M, Medeiros MC. Realized volatility: a review. Econometric Reviews. 2008;27(1–3):10–45. doi: 10.1080/07474930701853509

[34] Wei Y. Forecasting volatility of fuel oil futures in China: GARCH-type, SV or realized volatility models?. Physica A: Statistical Mechanics and its Applications. 2012;391(22):5546–56. doi: 10.1016/j.physa.2011.08.071

[35] Liu Q, Tao L, Wu W, Yu J. Short- and long-run business conditions and expected returns. Management Science. 2017;63(12):4137–57. doi: 10.1287/mnsc.2016.2552

[36] Leng N, Li J-C. Forecasting the crude oil prices based on econophysics and Bayesian approach. Physica A: Statistical Mechanics and its Applications. 2020;554:124663. doi: 10.1016/j.physa.2020.124663

[37] Francq C, Zakoïan J-M. Maximum likelihood estimation of pure GARCH and ARMA-GARCH processes. Bernoulli. 2004;10(4). doi: 10.3150/bj/1093265632

[38] Bates DS. Maximum likelihood estimation of latent affine processes. Rev Financ Stud. 2006;19(3):909–65. doi: 10.1093/rfs/hhj022

[39] Cipriani M, Guarino A, Uthemann A. Financial transaction taxes and the informational efficiency of financial markets: a structural estimation. Journal of Financial Economics. 2022;146(3):1044–72. doi: 10.1016/j.jfineco.2022.04.007

[40] Stroud JR, Johannes MS. Bayesian modeling and forecasting of 24-hour high-frequency volatility. Journal of the American Statistical Association. 2014;109(508):1368–84. doi: 10.1080/01621459.2014.937003

[41] Yu B, Zhong G-Y, Li J-C, Tang N-S. Bayesian estimation for stochastic dynamic equations via Fokker–Planck equation. Mod Phys Lett B. 2020;35(03):2150055. doi: 10.1142/s021798492150055x

[42] Wei Y, Wang Y, Huang D. Forecasting crude oil market volatility: further evidence using GARCH-class models. Energy Economics. 2010;32(6):1477–84. doi: 10.1016/j.eneco.2010.07.009

[43] Sun S, Wei Y, Tsui K-L, Wang S. Forecasting tourist arrivals with machine learning and internet search index. Tourism Management. 2019;70:1–10. doi: 10.1016/j.tourman.2018.07.010

[44] Hansen PR. A test for superior predictive ability. Journal of Business & Economic Statistics. 2005;23(4):365–80. doi: 10.1198/073500105000000063

[45] Wei Y, Nu-Tao Y. The predicting model of the volatility of China’s stock market and its SPA test. Journal of Financial Research. 2007;(07):138–50.

[46] Wei-Hua C. Forecasting volatility of Shanghai composite index with deep learning. Journal of Statistics and Information. 2018;33(05):99–106.

[47] Hansen PR, Lunde A. A forecast comparison of volatility models: does anything beat a GARCH(1,1)?. J of Applied Econometrics. 2005;20(7):873–89. doi: 10.1002/jae.800

[48] Bollerslev T, Engle RF, Nelson DB. ARCH models. Handbook of econometrics. 1994. p. 2959–3038.

[49] White H. A reality check for data snooping. Econometrica. 2000;68(5):1097–126. doi: 10.1111/1468-0262.00152

[50] Li J-C, Tao C, Li H-F. Dynamic forecasting performance and liquidity evaluation of financial market by econophysics and Bayesian methods. Physica A: Statistical Mechanics and its Applications. 2022;588:126546. doi: 10.1016/j.physa.2021.126546

[51] Wei Y. Multifractal volatility measure, its model and SPA test in financial market. Journal of Management Sciences in China. 2009;12:88–99.

